# Exon 10 CFTR gene mutation in male infertility

**Published:** 2012-07

**Authors:** Zohreh Hojati, Somaye Heidari, Majid Motovali-Bashi

**Affiliations:** *Genetics Division, Department of Biology, Faculty of Sciences, University of Isfahan, Isfahan, Iran.*

**Keywords:** *Infertility*, *Cystic fibrosis*, *Azoospermia*

## Abstract

**Background:** About 10% of infertilities with obstructive azoospermia are congenital and caused by CF gene mutations. M469I mutation was observed for the first time in Taiwanese patients. This mutation not only causes CF, but also may be the origin of infertility too.

**Objective:** In this study, we aimed in designing a rapid, reliable RFLP-PCR procedure for detection of M469I mutation. The correlation and association between M469I mutation with infertility was investigated in this study.

**Materials and Methods:** one hundred ten patients (90 non obstructive and 20 obstructive) and 60 normal individuals were considered in this study. M469I mutation was detected using RFLP-PCR. This technique was completely designed for M469I genotyping, for the first time in our study. Amplification of the region surrounding the mutation in exon 10 of *CFTR* gene was then performed. RFLP analysis was carried out using the *Nde*I restriction enzyme.

**Results:** All genomic DNA samples were genotyped successfully. M469I mutation was observed only in patients group. Therefore, genotype containing mutant allele (GT) has been detected only in the patients group. There was no significant correlation between GT and TT genotypes with infertility (p=0.437).

**Conclusion:** The M469I mutation has only been observed in Exon 10 *CFTR* gene of infertile patients, not in the control group. This mutation causes congenital bilateral absence of vaz deferens and finally infertility. This indicates a strong association between the M469I mutation and male infertility. Therefore, this is a CF-causing *CFTR* mutation that could be considered as a cause of infertility.

## Introduction

Inability to reproduce after a specified period of unprotected intercourse is named infertility. Reproductive sterility is permanent infertility. Infertility and sterility are then two different aspect of reproduction capability, the first describes as inability of couples to achieve clinically or biochemically recognizable pregnancy after at least one year of sexual intercourse while the later is complete and innate disabling in reproduction (1). 

Infertility can be primitive or secondary. About 15% of people in USA are infertile in their reproductive ages which include 10 million couples in that country. Either Male or female partner (even may be both) are responsible for infertility (1, 2). 

Congenital bilateral absence of the vas deferens (CBAVD) is a genital form of cystic fibrosis (CF) that is responsible for 2-6% of male infertility. The incidence of CF varies in different populations; therefore, the incidence of CBAVD will also vary in different populations. The spectrum and distribution conductance regulator (*CFTR)* gene mutations differ between CBAVD and CF patients and are comparable to control individuals. 

Combinations of particular alleles at several polymorphic loci yield insufficient functional CFTR protein. About ninety seven of CF men are congenital bilateral absence of vas deferens that blocks transfer of spermatozoa from testis or epididymal structure to external genital tract (3-5). The *CFTR *gene contains 27 exons encompassing 180 kb of DNA on chromosome band 7q31.2. The CFTR protein is a glycosylated transmembrane protein, which functions as a chloride channel. 

**Figure 1:**
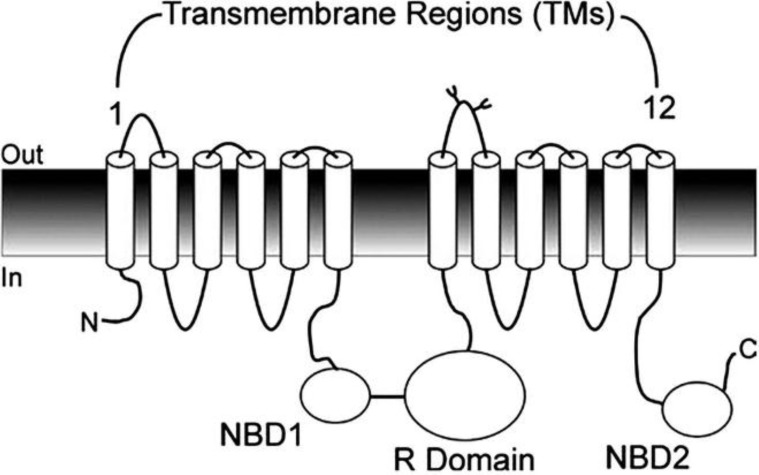
Topology of cystic fibrosis transmembrane conductance regulator (CFTR) protein.

The CFTR molecule is made up of 2 homologous repeats, each containing 6 transmembrane (TM) regions followed by an intracellular nucleotide-binding domain (NBD). These 2 halves are joined by an intracellular regulatory (R) domain (Figure 1) (3-8)*. *CFTR transcripts are confined to post meiotic round spermatids. During this development stage, spermatozoa are formed from haploid spermatids. 

Nucleus condensation **and** decrease in cytoplasm volume, which are thought to be caused by reduction of intracellular water content, occur in this phase. Maximal *CFTR* expression precedes this stage. Mammalian sperm for fertilizing need one process called capacitation that is related with increasing in intracellular pH and hyperpolarization of sperm’s membrane. These changes are depended on extracellular HCO_3_^-^. CFTR is channels that conduct Cl^-^ and HCO_3_^-^ transportation and mutation in this gene cause non-capacitation of sperm. 

These situations ultimately cause male infertility (7). Most of *CFTR *mutations were in exons 2-5, 7, 9-13, 17b and 19-21, which encoding transmembrane domain (TMD) and nucleotide domain (NBD) (8-10). Different varieties of CF-causing *CFTR* mutations have been identified so far (more than 1500). Most of them are point mutations. A CF patient can either carry an identical *CFTR* mutation on both *CFTR* alleles or 2 different *CFTR* mutations on both *CFTR* alleles. The distribution of *CFTR* mutations varies between different ethnic populations. It has been found that the F508del mutation has the highest frequency (about 70%) in Northern European populations, while lower frequencies are observed for this mutation in Southern European populations. 

Besides F508del, other common mutations exist in most populations, within frequencies of about one percent (9). Therefore for a given population, ethnic-specific mutations that reach frequencies of about 1-2% might exist. The association between some of these *CFTR* mutations and male infertility have already been detected like ΔF508, M470V, ΔI507, N1303K (11-13). About 10 percent of infertilities with obstructive azoospermia are congenital and caused by CF gene mutations. Here, a procedure based on PCR was designed for rapid detection of M469I mutation. 

This mutation not only causes CF, but also may be the origin of infertility too, therefore the association between this mutation in cystic fibrosis transmembrane conductance regulator gene and male infertility was investigated in this study. 

## Materials and methods

**Patients**

This study is a case-control study. Patients were infertile men who approached in Isfahan Infertility Center and Sari Saint Mary Infertility Center. Detailed questionnaires, including clinical and family history, were initially collected. Written consent was then given to collect blood samples. Diagnosis of CBAVD patients was initially performed by physical examination, and transabdominal/ rectal ultrasonography. 

These observations were subsequently confirmed as azoospermia using the semen analysis. In this study patients with semen volume below 1.5 ml (cytobiochemical characteristics) were considered for this study (8 and 14). One hundred azoospermia’s men are included in this study. Samples were 23-48 years old (mean age 31.5). These patients are divided in two groups: 80 non obstructive and 20 obstructive (CBAVD) patients. Sixty normal men were also studied as the control group. These men were selected voluntarily from healthy fathers.

**Total DNA isolation**

Two ml blood samples were collected from patients and control persons. Tubes contain EDTA was used here in order to prevention of blood clotting. The collected blood samples were gently shaken and kept on ice. The salting-out procedure was used for genomic DNA extraction. All DNA samples were diluted to the same concentration (50 ng/µl). 

**Primer design**

Sequence of exon 10 *CFTR* gene was achieved from NCBI (http://www.ncbi.nlm.nih.gov). The location of the M469I mutation was also determined in this exon. Specific primers were designed in order to amplify the region surrounding that specified mutation (using Oligo6 ® and CLC softwares). The sequences of primers are shown in table I. 

There is no cut site for the *Nde*I restriction enzyme in that region. The polymorphism that produced by the M469I mutation also does not produce any cut site for the *Nde*I. The forward primer was subjected to a couple of bases modification. The amplification of exon 10 will produce a fragment that contains an *Nde*I cut site, if there is no M469I mutation in this region. In the presence of M469I polymorphism, the amplified product has no cut site for the *Nde*I. A 240bp DNA fragment will be amplified by the PCR. *Nde*I cuts this fragment at position 25 and produce a 25bp and a 215bp fragments (Figure 1).

**RFLP-PCR**

The region in proximity of the M469I mutation was amplified by specific designed primers (Table I), using Ependorf PCR machine (Germany). The amplified fragment was subjected to digestion with *Nde*I (from Fermentase Corporation) using its specific buffer. The PCR products and cut fragments were separated using a 2% agarose gel.

**Statistical analysis**

Three GG, GT, TT genotypes for M469I polymorphism was studied here by RFLP technique. The ^2^ test was used here to ascertain the variation in frequencies of these genotypes between patients and controls.

## Results

The PCR was carried out using the isolated DNA as template and designed primers, CF-M469I-F and CF-M469I-R. A 240bp amplified fragment was observed following gel electrophoresis. RFLP-PCR was performed by digestion of the amplified fragment with *Nde*I restriction enzyme. All the 60 amplified fragments which obtained from the control persons were completely digested by *Nde*I and produced two fragments with different length (25bp and 215bp). The absence of mentioned mutation (GG genotype) causes full enzymatic digestion of the amplified fragment in the persons of the control group. 

Gel electrophoresis analyses were also performed on the *Nde*I cut amplified fragments obtained from all the azoospermia patients group too. Gel electrophoresis analyses of *Nde*I cut amplified fragments have revealed the existence of a sample containing cut and uncut fragments. So this azoospermia patient carries two non-identical alleles, including one T that indicates the M469I mutated allele and one G that indicates the normal or wild type allele. This situation indicates the existence of a heterozygous patient with GT genotype. 

**Figure 2:**
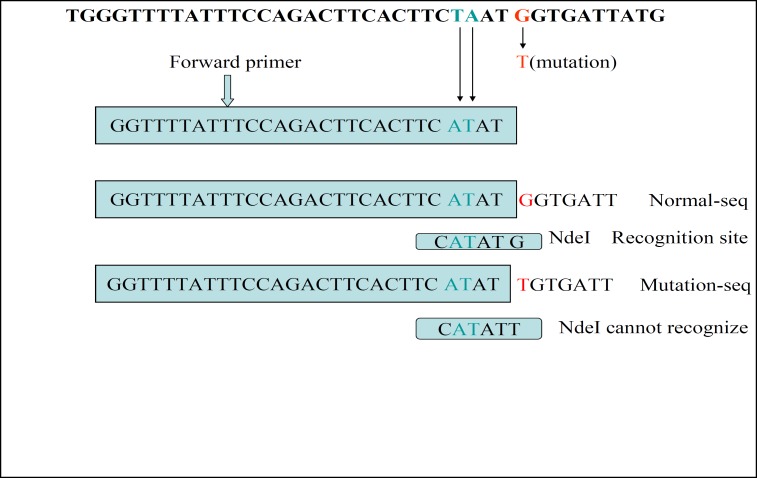
Schematic representation of the exon10 amplified region

The positions of created bands (for a normal and a heterozygous sample) are illustrated in Figure 2. Therefore Genotypes containing mutant allele (GT) have been detected only in the patients group (1%). The M469I mutation was studied here by RFLP-PCR. The GG, GT, TT genotypes of this mutation were observed in 100%, 0%, 0% of the control group and in 99%, 1%, 0% patients (Table II). The statistical analysis has shown no correlation between GT and TT genotypes with infertility (OR=0.990, 95% confidence interval CI=0.971-1.010, p=0.437).

**Table I:** Frequencies of G/T genotypes in control and patient groups

**Genotype**	**Controls n(%)**	**Patients n(%)**	
GG	60 (100%)	99 (99%)
GT	0 (0%)	1 (1%)
TT	0 (0%)	0 (0%)

Three different genotypes were analyzed in this study; these were included GG, GT and TT. The frequencies of these genotypes are mentioned here.

CFTR comprises 12 TM regions (organized into 2 groups of 6), 2 intracellular nucleotide-binding domains (NBDs), and the intracellular regulatory (R) domain (9).

The modified region on the forward primer is shown here. This designation was done in such a way that produces a recognition site for the *Nde*I restriction enzyme. The amplified fragment (from the normal person) contains this cut site. So the *Nde*I restriction enzyme is able to cut it completely. This *Nde*I enzyme cannot digest the amplified fragment from the mutant persons.

**Figure 3:**
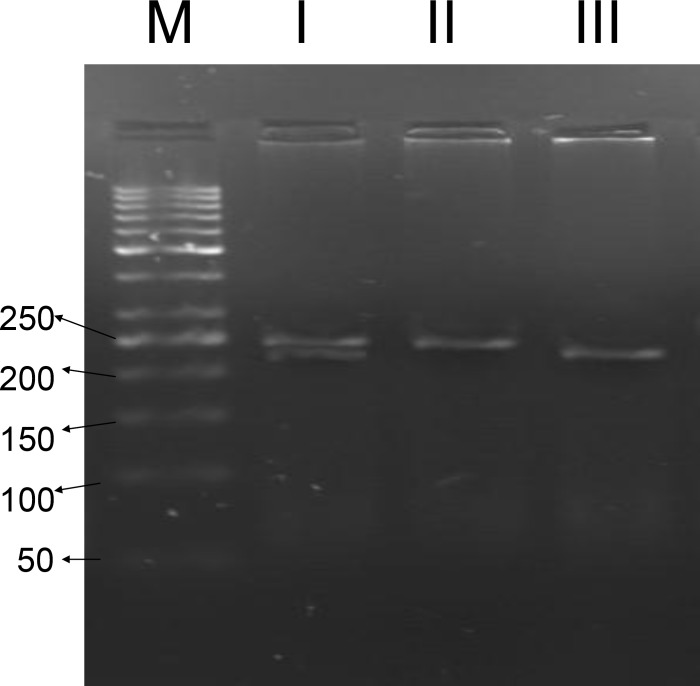
M469I SNP detection in exon 10 of *CFTR* gene using RFLP-PCR reaction

A set of specific designed primers (CF-M469I-F and CF-M469I-R) were used for amplification of some part of exon 10 of the *CFTR* gene. The 240 bp amplified fragment was subjected to digestion with *NdeI. *The resultant fragments were separated on a 2.5% agarose gel. Different genotypes are identified according to the produced patter of the bands on the gel. A 50 bp DNA ladder was used as marker. I heterozygote infertile patient (GT: M469I), II uncut PCR product, III a normal homozygote GG person. Numbers are in base pair.

## Discussion

There are different varieties of mutations in NBDI that the associations of them with infertility haven't been determined yet. M469I mutation that occurs in NBD1 was observed for the first time in Taiwanese patients in 2005. They included 36 CBAVD patients in their study; one of them was detected to have M469I mutation. He was heterozygote for this mutation (15 and 16). They used Temporal Temperature Gradient Electrophoresis (TTGE) technique in order to screen the polymorphisms and mutations. 

TTGE analysis reveals polymorphism by shifting the produced bands on polyacrylamide gel. The DNA fragments that showed abnormal banding patterns on TTGE analysis were sequenced in order to determine the actual sequences of the nucleotides in this region of the gene (17). 

This technique is time-consuming expensive and requires further sequencing of the samples to reveal the actual nature of the polymorphism. On the other hand recently Bartoszewski *et al* has demonstrated that the DeltaF508 mutation alters the secondary structure of the CFTR mRNA. This DeltaF508 CFTR mRNA cannot be translated efficiently. So the translational rate of this "mis-folded" mRNA is reduced dramatically. Then, mutation may also contribute to the differences observed in the symptoms of various DeltaF508 homozygous CF patients (18). Designing simple, rapid and inexpensive procedures for identification of mutations, could improve the molecular detection of different varieties of diseases, especially infertility. Recently 3 novel intragenic polymorphic repeats (IVS3polyA, IVS4polyA, and IVS10CA repeats) were found in the *CFTR* gene, using a simplified procedure based on the PCR (19). 

Two uncommon variants have been identified in year 2010 that may be considered as a novel cause of CBAVD (20). More investigation is then required in this regard. In this study an RFLP-PCR procedure was designed in order to be able to screen a large group of patients for a specific and pre-determined mutation. This procedure is quite rapid and reliable. The purpose of this study was investigation of the existence of M469I mutation in Iran's population. This finding could reveal association of this mutation with male infertility. Therefore, recognition and identification of mutations are very important, not only in genetic counseling but also in ascertaining the outcome of the disease. 

Here we screened one hundred azoospermia infertile male for the existence of the M469I mutation. About one percent of the infertile population under investigation has shown the heterozygous genotype (GT). No mutant allele (T) was observed in individuals of control group. There was no significant correlation between GT and TT genotypes with infertility (p=0.437) (OR=0.990, 95% confidence interval CI=0.971-1.010, p=0.437). Scientist screened 36 Taiwanese patients for this mutation (using TTGE technique) and they have also found one heterozygote person. 

Although the statistical analysis has shown no correlation between GT and TT genotypes with infertility (possibly because of the patients number that have studied), but because this mutation has only been detected in patients group, it still could be considered as a risk factor and molecular marker for fertility assessment. When M469I mutation occurs in nucleotide binding domain of CFTR gene, it damages the normal structure of transmembrane protein. This abnormality causes congenital bilateral absence of vaz deferens and finally infertility. This indicates a strong association between the M469I mutation and male infertility. 

It is clearly concluded from this study that as long as this mutation occurs or exist in a person, he should be considered as an infertile person. This mutation should be used in panel of mutations for screening in couples that use assisted reproductive techniques for treatment of infertility, such as Intra Cytoplasmic Sperm Injection (ICSI) and IVF (21). So M469I could be used in genetic counseling and pre-implantation genetic detection, to prevent from further fertility recurrence in successive generations.
